# Efficacy of a dietary supplement in dogs with osteoarthritis: A randomized placebo-controlled, double-blind clinical trial

**DOI:** 10.1371/journal.pone.0263971

**Published:** 2022-02-16

**Authors:** Elisa Martello, Mauro Bigliati, Raffaella Adami, Elena Biasibetti, Donal Bisanzio, Giorgia Meineri, Natascia Bruni

**Affiliations:** 1 Division of Epidemiology and Public Health, School of Medicine, University of Nottingham, Nottingham, United Kingdom; 2 Ambulatorio Vernazza, Torino, Italy; 3 Candioli Pharma S.r.l., Beinasco (TO), Italy; 4 Istituto Zooprofilattico Sperimentale del Piemonte, Liguria e Valle d’Aosta, Torino, Italy; 5 RTI International, Washington, DC, United States of America; 6 Department of Veterinary Science, University of Turin, Grugliasco (TO), Italy; Michigan State University, UNITED STATES

## Abstract

This study is a randomized, placebo-controlled, double-blinded trial performed to investigate the effects of a dietary supplement containing a mixture of *Boswellia serrata* Roxb., chlorophyll, green tea extract, glucosamine, chondroitin sulfate, hyaluronic acid, and further in the manuscript: non-hydrolised type II collagen in dogs with osteoarthritis (OA). A total of 40 dogs were enrolled in the study, they were randomly divided in control (CTR) and treatment (TRT) groups. The TRT group received the dietary supplement for 60 days. The CTR group received a placebo for the same number of days. All the subjects had veterinary evaluations during the trial and owners were requested to fill in questionnaires on chronic pain using the Helsinki Chronic Pain Index. The product was easy to administer and no side effects were reported. Combining results from veterinarian and owner evaluations, the tested product proved to be significantly beneficial in alleviating pain and in reducing the clinical signs in dogs with OA.

## 1. Introduction

Osteoarthritis (OA) is a common condition in humans and pets. It is characterized by a change in tissues of synovial joints, with chronic pain and disability as a result of the articular cartilage deterioration. In dogs, trauma, genetic predisposition, and excessive exercise could be common causes of OA; while the breed, gender, animal size and overweight are known risk factors [1, 2]. Common findings in dogs diagnosed with OA are: hypertrophy of bone at the margins, degeneration of the cartilage, and changes in the synovial membranes. Animal examination performed by veterinary specialists using radiographs, clinical signs, type and degree of lameness, and OA risk factors can help predicting the risk of joint degradation [3]. In veterinary medicine assessing and evaluating the degree of pain is very difficult. In literature, several questionnaires have been developed and validated to obtain valid and comparable information from animals’ owners on signs of acute and chronic pain and to highlight changes of attitude and behavior of their affected animals over time [4–6]. In particular, the Helsinki Chronic Pain Index (HCPI) is a questionnaire validated for chronic pain associated with OA in dogs. This includes 11 questions related to mood, lameness, and willingness to move, play, and jump [5, 7, 8]. Unfortunately, when OA is clinically evident it cannot be cured, with pain and associated loss of joint function as signs that interfere with the normal life of affected humans and animals. The main aim of a clinician is to reduce the chronic pain improving the patient wellbeing. Drugs like nonsteroidal anti-inflammatory drugs (NSAIDs) could be of some help reducing symptoms of OA, but side effects have been reported by veterinarians [9–11]. Alternatively or in addition to these, physiotherapy, hydrotherapy, and acupuncture are common forms of treatment [12]. Lastly, dietary supplements and traditional herbal products are increasingly used for treatment of pain. Chondroprotective and anti-inflammatory properties of these products have also been reported [8, 13]. For example, a common ingredient used to regulate the collagen synthesis in cartilage providing anti-inflammatory effects is glucosamine. Glucosamine is often associated to chondroitin sulfate, and inhibits destructive enzymes in joint fluid and cartilage [14]. Other authors studied the use for OA management like hyaluronic acid and undenatured type II collagen [15–17]. Natural ingredients of plantal origin like *Boswellia serrata Roxb* [13, 18], chlorophyll [18–20] and green tea extract [19, 21] have been reported to be used in humans and animals as anti-inflammatory.

In this study, we investigated the effect on chronic pain and clinical signs of a supplement (*Confis Ultra*, Candioli s.r.l., Italy) containing a mixture of *Boswellia serrata Roxb*., chlorophyll and green tea extract in addition to some chondroprotectants (Chondroitin sulfate, glucosamine and hyaluronic acid) on dogs affected by OA. Clinical trials were already performed with this supplement on dogs with OA with satisfactory effects [22] but these rigorous randomized controlled trials could be useful to strengthen the previous results.

## 2. Materials and methods

### 2.1. Animals included and recruitment

Dogs were included when client-owned >6 months of age, weighting 10–60 kg, with body condition score 2–4 (on a scale of 1–5) and muscle condition score (MCS) (WSAVA Global Nutrition Committee) between “normal” and “moderate loss”, and with radiographic signs of OA on their elbow, knee, or hip. The protocol provided the exclusion of dogs with acute pain, signs of recent trauma or surgery (in the previous 6 months), neurological conditions, treatment with corticosteroids or NSAIDs or other supplements 14 days before the beginning of the study, allergy to any of the ingredients included in the tested supplement.

### 2.2. Study design and the dietary supplement

This study was designed as a randomized placebo-controlled, double-blinded clinical trial. A total of 40 dogs were randomly assigned to two groups: Control (CTR, n = 20) and Treated (TRT, n = 20) groups. Animals belonging to the control CTR group were administered a placebo (ingredients listed in Table 1), while the TRT group were administered the tested supplement (*Confis Ultra*, Candioli Pharma S.r.l., Italy).

**Table 1:** Composition of the placebo used during the study.

COMPOSITION	% in 2.0 gr of tablet
Maltodextrin	68.5
Flour od Guar seeds	5
Microcrystalline cellulose type II	13
Magnesium stearate	1.5
Dicalcium phosphate dihydrate	5
Appetite stimulants (D’Tech 8P)	7
**TOTAL**	***100***

Details of the ingredients included in the tablets are listed in Table 2.

**Table 2:** Composition of the feed supplement used during the study.

COMPOSITION	% in 2.0 gr of tablet
Hyaluronic acid	0.9
Chondroitin sulfate	15
Glucosamine hydrochloride	25
Collagen type II-not hydrolyzed	0.2
FLEXIDE^®^ (*Camellia sinensis* (L.) O. Kuntze—green tea extract, *Boswellia serrata* Roxb. ex Colebr., Copper complexes of chlorophylls (E141)	3.5
Appetite stimulants (D’Tech 8P)	6.7
Technological additives (antioxidants, emulsifiers, stabilizing agents)	48.7
**TOTAL**	***100***

A dose of one tablet (2 gr) per 10 kg of body weight (BW) was orally administered once daily for six weeks. The product belongs to the category “feed supplement” in compliance to the REGULATION (EC) No 767/2009 of the European Parliament and Council (13 July 2009) related to the placing on the market and to the use of feed, amending the European Parliament and Council Regulation (EC) No 1831/2003 and repealing the Council Directive 79/373/EEC, Commission Directive 80/511/EEC, Council Directives 82/471/EEC, 83/228/EEC, 93/74/EEC, 93/113/EC and 96/25/EC and Commission Decision 2004/217/EC.

The dogs’ owners were informed about the purpose and the design of the study, and signed a written informed consent. All procedures, treatments and animal care were in compliance with the guidelines of the Italian Ministry of Health for the care and use of animals (D.L. 4 March 2014 n. 26 and D.L. 27 January 1992 n.116) and UE (Directive 86/609/CEE) and the use of supplements was regulated by the Regulation (EC) No 767/2009. The experimental protocol was designed according to the guidelines of the current European and Italian laws on the protection of animals used for scientific purposes (Directive 2010/63/EU, put into force in Italy with D.L. 2014/26). Furthermore, the experimental protocol was approved by the Ethical Committee of the Department of Veterinary Science (DVS) of the University of Turin (Italy) (prot. n. 709–17/03/2021).

### 2.3. Veterinary and owner evaluations

Dogs were subject to different veterinary and owner evaluations at different time points during the observational period: baseline (T0), then after 40 (T1) and after 60 (T2) days.

Data on sex, age, breed, body weight, body condition score (BCS, 9 points scoring system [23]), were recorded at T0. A radiological assessment was carried out at the first visit with the sole objective to confirm the diagnosis of OA to include the animal in the study, as no significant changes in the radiographic findings are expected to be found over the 60 days period.

The same veterinarian performed a complete blood analysis at T0 and T2: complete blood count (CBC) and serum biochemical analysis.

During each of the three visits, a veterinarian experienced in orthopedics recorded clinical signs of osteoarthritis using a scale of grades 0–4 (0 = no signs; 1 = minor disability, first-degree cold lameness that disappears after a short activity; 2 = moderate disability, first-degree cold lameness that disappears after a moderate activity; 3 = severe disability, second-degree lameness that continues after a moderate and prolonged activity; 4 = loss of movement, third-degree permanent lameness, independent from the type of activity) at T0, T1, and T2. Then, the owner assessed the chronic pain using an Italian adapted version of the validated Helsinki Chronic Pain Index (HCPI), already utilised in another study [22], that is a functional behavior-based owner questionnaire for veterinary use, based on a previous study published by Canapp and colleagues [8]. The questionnaires on chronic pain index (HCPI) in dogs were completed by all owners at T0, T1 and T2.

### 2.4. Statistical analysis

Statistical analysis was performed using the R language [24]. Descriptive statistics were used to explore the data. The T-test and Mann-Whitney U test was performed to see differences in the mean baseline parameters (T0) between TRT and CTR groups respectively on BW and BCS.

A Mann-Whitney U test was performed to test the median difference in HCPI scores and clinical signs of OA between the two groups at different time points (T0, T1, T2). Wilcoxon signed-rank test was performed to demonstrate differences in HCPI as well as clinical signs of OA scores within groups between T0, T1 and T2.

The effect of the supplement on HCPI and degree of clinical signs of OA recorded during the study was investigated using a regression model. The model was built as a generalized linear mixed model (GLMM) with Gaussian likelihood. The model included a non-linear variable describing the link between each sampling time within and between the CTR and TRT group, sex, weight, and BCS. The identification of the subject was included in the model as a random effect in order to account for repeated measurements and the heterogeneity of individuals.

## 3. Results

All the 40 dogs enrolled in the study completed the trial. The owners reported good palatability and ease of administration of the tested dietary supplement. No adverse effects (vomiting, diarrhea, anorexia), monitored daily by the owners, were observed during the whole study period in any of the dogs.

The median age of the dogs was 8.2 years, with dogs belonging to the CTR group ranging from 4 to 12 (mean 8.3± 2.4 SD) and to the TRT group from 3 to 11 (mean 8.1± 2.1 SD). In the CTR group 11 dogs were females and 9 males, while in the TRT group 9 dogs were females and 11 males. A total of 14 dogs were mixed breed (n = 7 CTR; n = 6 TRT), one was Boxer (n = 1 TRT), five were Labrador (n = 2 CTR; n = 3 TRT), three were German Sheperd (n = 1 CTR; n = 2 TRT), three were Golden Retriever (n = 2 CTR; n = 1 TRT), two were Syberian Husky (n = 1 CTR; n = 1 TRT), two were Rottweiler (n = 1 CTR; n = 1 TRT), one was American Staffordshire (n = 1 TRT), one was Belgian sheperd Malinois (n = 1 TRT), one was Belgian shepherd Tervueren (n = 1 TRT), one was English Setter (n = 1 TRT), one was Epagneul Breton (n = 1 TRT), one was Dogue De Bordeaux (n = 1 CTR), one was Lagotto Romagnolo (n = 1 CTR), one was Bullmastiff (n = 1 CTR), one was Weimaraner (n = 1 CTR), and one was Flat-Coated Retriever (n = 1 CTR).

The radiographic examination at the baseline (T0) was used to confirm OA conditions in all dogs included and to highlight the involvement of one or more joints. More specifically, the joints affected by OA (n = number of cases) are listed here: knee unilateral (n = 4), hip unilateral (n = 7), hip bilateral (n = 6), elbow and hip (n = 4), elbow unilateral (n = 11), and elbow bilateral (n = 8).

The complete blood analysis at T0 and T2 highlighted all values within the normal ranges both times for all dogs, so we considered the product safe. This also allowed us to include only patients without current pathologies.

At the time of the enrolment no significant difference in BW and BCS was reported between groups (p = 0.769 and p = 0.586, Table 3).

**Table 3:** Body weight (BW) and Body condition score (BCS) in the two groups (control-CTR and treatment-TRT) at the baseline of the study (T0).

**Body weight (BW)**	**CTR (n = 20)**	**TRT (n = 20)**	**Student T-Test (P-value)**
Mean (Kg)	29.9	29.1	0.769
Std. Deviation (Kg)	8.1	9	
Minimum (Kg)	18	14	
Maximum (Kg)	47	55	
**Body Condition Score (BCS)**	**CTR (n = 20)**	**TRT (n = 20)**	**Mann-Whitney U test (P-value)**
Median	3	3	0.586
Q1, Q2	3, 3.25	3, 4	

Data are expressed as mean and standard deviation for the BW; median, first (Q1), and third (Q3) quartiles for BCS. Differences between groups are calculated using Student T-Test (BW) and Mann-Whitney U test (BCS).

**Fig 1:**
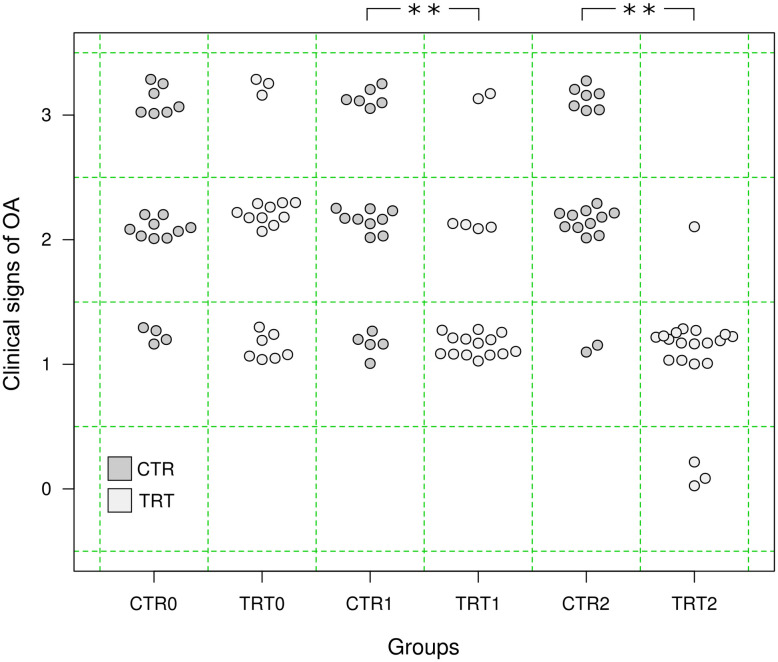
Comparison of clinical signs of OA between CRT and TRT groups at three time points (T0, T1, T2), **p<0.01, Mann-Whitney U test.

The veterinarian evaluated the animal clinical signs of OA progression grading it at T0, then at T1 and T2. Specifically, in the CTR group seven animals started with grade 3 (35%), nine animals with grade 2 (45%), and four with grade 1 (20%); while in the TRT group three animals started with grade 3 (15%), ten animals with grade 2 (50%), and seven with grade 1 (35%). During the period between T0 and T2, six animals in the TRT group remained with the same score, while the others (n = 14) improved their condition. In the CTR group, in three cases the clinical signs got worse (score 1 to 2 n = 2; score 2 to 3 n = 1), one improved (score 3 to 2) and the other 16 remained the same. A significant improvement of signs of OA was recorded in the TRT group at T1 (Wilcoxon signed-rank test, p<0.001) and T2 (Wilcoxon signed-rank test, p<0.001), while no differences were found in the CTR group overtime (Wilcoxon signed-rank test, p>0.05). No significant difference was found in the median clinical signs scores between groups at T0 (Mann-Whitney U test, U = 251.5, p = 0.14), while at T1 (U = 293, p<0.01) and T2 (U = 376.5, p<0.01) the difference was significant (Fig 1).

As resulted from the GLMM, an improvement of clinical signs of OA was significant only at T2 in the TRT group (Table 4).

**Table 4:** Effect (β) of time on signs of OA scores recorded in the two groups (CTR and TRT) estimated by the performed GLMM.

Time	Group	Effect β (95% CI)
T0	CTR	0.3 (-0.1; 0.8)
	TRT	0.1 (-0.5; 0.6)
T1	CTR	0.2 (-0.3; 0.7)
	TRT	-0.3 (-0.8; 0.2)
T2	CTR	0.4 (-0.1; 0.9)
	TRT	-0.8 (-1.3; -0.3) *

*p<0.05.

**Fig 2:**
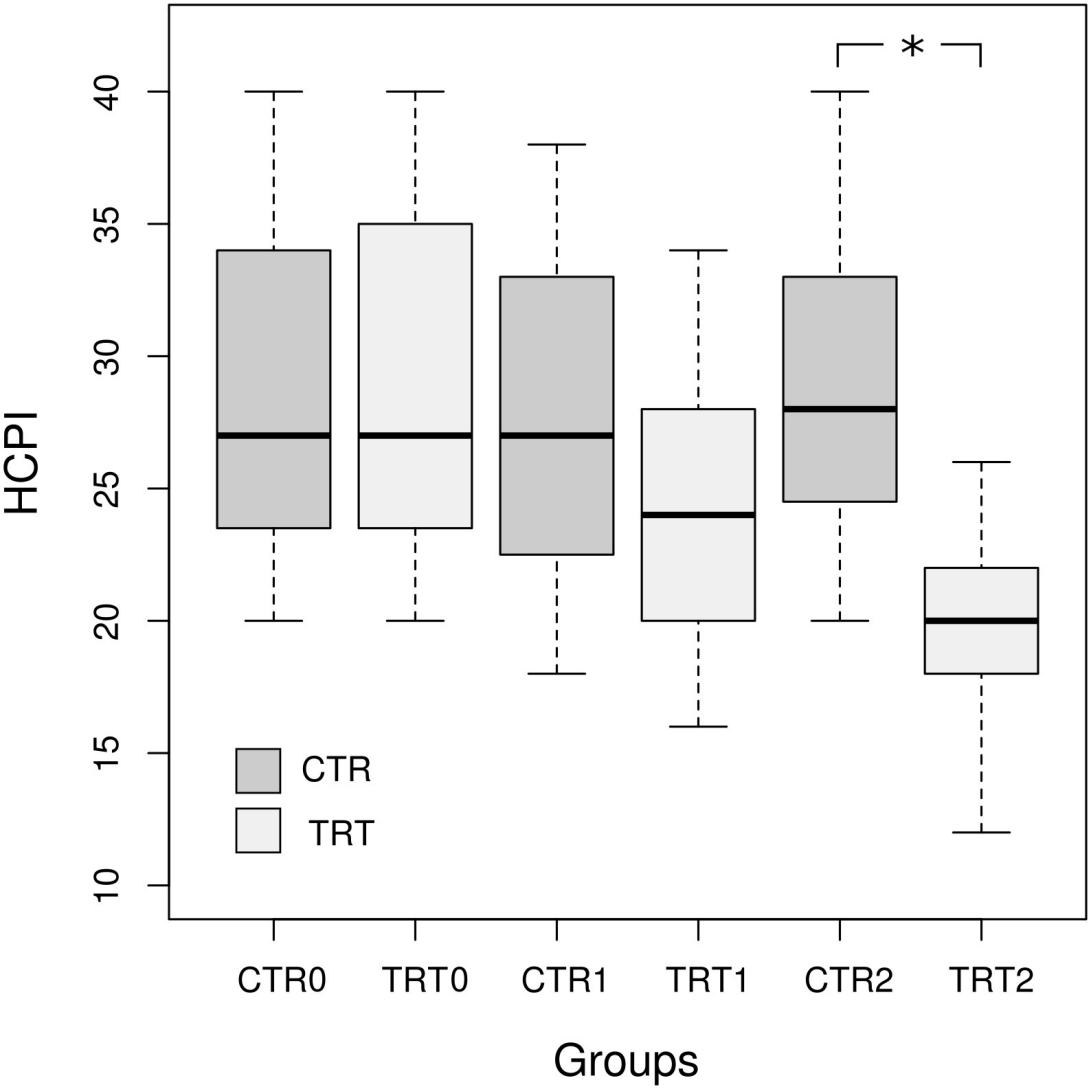
Comparison of HCPI scores between CRT and TRT groups at three time points (T0, T1, T2), * p<0.05, Mann-Whitney U test.

The questionnaires on chronic pain index (HCPI) in dogs were completed by all owners at T0, T1 and T2. No significant differences in median HCPI scores between groups at T0 (U = 196, p = 0.924, Mann-Whitney U test) and T1 was identified (U = 261.5, p = 0.097, Mann-Whitney U test), while a significant difference was reported at the end of the study (U = 373.5, p<0.001, Mann-Whitney U test), when the TRT group scores were significantly lower than the CTR group (Fig 2).

A significant decrease in the median score from the beginning to the end of the study for the TRT group was recorded (p<0.001, Wilcoxon signed-rank test). For the CTR group, where the median scores remained similar for the entire duration of the study, non-significant difference was detected (p = 0.162, Wilcoxon signed-rank test).

As resulted from the GLMM, a decrease of HCPI score was significant only at T2 in the TRT group (Table 5).

**Table 5:** Effect (β) of time on HCPI scores recorded in the two groups (CTR and TRT) estimated by the performed GLMM.

Time	Group	Effect β (95% CI)
T0	CTR	1.6 (-2.4; 6.1)
	TRT	3.1 (-1.6; 7.1)
T1	CTR	0.9 (-2.9; 5.3)
	TRT	-1.7 (-6.2; 2.3)
T2	CTR	1.9 (-1.8; 6.4)
	TRT	-6.2 (-10.8; -2.2) *

*p<0.05.

Raw data are provided in S1 Data.

## 4. Discussion

Osteoarthritis is a chronic debilitating disease commonly diagnosed by veterinarians in dogs. The use of long-term treatments with traditional drugs is the elective choice among clinicians, but most of times [25] it could lead to a high risk of developing side effects in patients. An alternative approach is to use natural products for treating OA in both humans and animals with different results as reported in literature [26–28].

In this study, a dietary supplement was tested on dogs with OA for a period of 6 weeks administration, during a 60-day trial. No adverse effects were reported, confirming the safety and tolerability of the product.

Our study is in agreement with other ones, where diet supplements are well tolerated and absorbed quickly [22, 29, 30]. For safety reasons, the list of materials for animal nutrition placed on the market and in the present supplement is authorized by the EU Commission with the technical support of EFSA, and then considered safe.

With regards to the clinical efficacy (clinical signs of OA), the product resulted effective after 60 days of supplement administration taking into account sex, weight, and BCS. Interestingly, the owners’ opinions through the HCPI confirmed that also the animal’s mood, lameness, and willingness to move, play, and jump significantly ameliorated after 60 days of treatment in the TRT group taking into account the sex, weight, and BCS. This was also reported in a previous study on dogs [22].

Beside the interesting findings of this trial, some limitations have to be reported: the disease nature itself, the level of OA potentially conditioned by the environment, the different clinical manifestation and the etiopathogenesis [5]. In fact, it cannot be excluded that in our study these factors could have influenced the final results related to both placebo and treated groups.

## 5. Conclusions

Our results support the use of a dietary supplement as a potentially efficacious treatment in cases with different clinical conditions, level of chronic pain and joint involvement. The tested product was beneficial in alleviating pain and reducing clinical signs in dogs with OA. Further studies comparing its effects to common drug therapies, such as NSAIDS, are warranted.

## Supporting information

S1 Data (CSV)
